# Mechanisms and functions of endocytosis in T cells

**DOI:** 10.1186/s12964-021-00766-3

**Published:** 2021-09-09

**Authors:** John C. Charpentier, Philip D. King

**Affiliations:** Department of Microbiology and Immunology, University of Michigan Medical School, 6606 Med Sci II, 1150 West Medical Center Drive, Ann Arbor, MI 48109-5620 USA

**Keywords:** Endocytosis, Macropinocytosis, T lymphocytes, TCR signaling, Cell growth

## Abstract

Once thought of primarily as a means to neutralize pathogens or to facilitate feeding, endocytosis is now known to regulate a wide range of eukaryotic cell processes. Among these are regulation of signal transduction, mitosis, lipid homeostasis, and directed migration, among others. Less well-appreciated are the roles various forms of endocytosis plays in regulating αβ and, especially, γδ T cell functions, such as T cell receptor signaling, antigen discovery by trogocytosis, and activated cell growth. Herein we examine the contribution of both clathrin-mediated and clathrin-independent mechanisms of endocytosis to T cell biology.

**Video Abstract**

**Supplementary Information:**

The online version contains supplementary material available at 10.1186/s12964-021-00766-3.

## Background

Endocytosis, the generation of internal membranes from the plasma membrane by invagination and vesicle scission, facilitates a range of diverse cellular processes in eukaryotes. In addition to enabling the internalization of extracellular macromolecules, endocytosis permits the compartmentalization of chemistry within cells. Co-evolution of endocytosis and cellular endosymbiosis, the state of one cell living mutualistically within another, may have significantly contributed to the complexity of eukaryotic cells [1]. Functions regulated at least in part by endocytosis include: signal transduction, membrane composition, mitosis, adhesion, lipid homeostasis, motility, and cell morphogenesis. Distinct forms of endocytosis have evolved in eukaryotes, with clathrin-mediated endocytosis (CME) being the most well-described and universal type. Other forms are limited to and adapted for specific cell types or lineage states.

Whereas endocytic regulation of some cellular functions, such as immune surveillance, has been extensively described in some immune cell types (such as dendritic cells and macrophages), its functional importance in T cells has been less appreciated. This review will discuss the role of endocytosis in the regulation of T cell function.

## Forms of endocytosis

### Clathrin-mediated endocytosis

Endocytic pathways are often broadly classified by their dependence on the hexamer protein clathrin. This is in part due to the historical primacy of the characterization of clathrin-mediated endocytosis (CME) in 1976 but also in acknowledgement of its role as the primary endocytic route for cellular housekeeping functions [65]. In CME, the assembly of clathrin triskelions on spherical membrane buds drives the formation of clathrin-coated pits (CCPs) 60–120 nm in diameter [66]. CCPs progress through a series of well-defined morphological intermediates to form clathrin-coated vesicles (CCVs) upon scission from the plasma membrane. Post-scission, the clathrin assemblies disintegrate, additional machinery is removed by uncoating factors, and uncoated vesicles deliver their contents to endosomes by fusion [67]. CCVs can be further classified by the differential recruitment of over 50 adaptor and accessory proteins, as well as by the identity of their lipid and protein cargoes [66].

For many years, the term “receptor-mediated endocytosis” was used synonymously with CME. It is now appreciated, however, that removal of many plasmalemmal receptors is accomplished by multiple mechanisms that do not require clathrin. Consequently, this usage is discouraged and a more descriptive schema—classification of endocytic routes by the identity of vesicular membrane components and cargoes—has been adopted.

### Clathrin-independent mechanisms of endocytosis

Some cellular functions, such as response to high intensity stimuli and directed migration, require rapid endocytosis of large patches of membrane. These events require membrane fluxes on the millisecond-to-second scale, which CME is not sufficient for [68]. In recent decades, a number of mechanisms of clathrin-independent endocytosis (CIE) have been discovered and characterized, some of which enable rapid, bulk internalization of membrane or otherwise facilitate acute responses. Relative to CME, flux through CIE pathways accounts for only a small proportion of endocytic events in mammalian cells [69]. Our present knowledge of CIE is chiefly limited by the lack of validated, path-specific molecular determinants and cargoes, as well as the existence of shared machinery between pathways and these factors confound interpretation of experimental results. Nevertheless, salient features of each form have been experimentally elucidated.

Building on the classification of Doherty and McMahon (2009), CIE includes: caveolae-dependent endocytosis, clathrin-independent carrier/GPI-AP-enriched early endosomal compartment (CLIC/GEEC) pathway endocytosis, flotillin-dependent endocytosis, interleukin 2 receptor beta (IL-2Rβ) pathway endocytosis, Arf6-dependent endocytosis, phagocytosis, macropinocytosis, fast endophilin-mediated endocytosis, activity-dependent bulk endocytosis (ADBE), ultra-fast endocytosis (UFE), and massive endocytosis (MEND) [70]. Each of these forms of endocytosis, the essential features of which are summarized in Table 1, will be discussed briefly.

**Table 1:** Modes of endocytosis and their salient features

	Actin-dependent	Scale (vesicle diameter)	Canonical cargoes	Cholesterol-dependent	Dynamin-dependent	Cell type first described in
Clathrin-dependent endocytosis	Depends on cell type [2]	35–200 nm [3]	Tfr [4]	Yes [5]	Yes [6, 7]	*A. aegypti* oocytes [8]
Caveolae-dependent endocytosis	Yes [9–11]	50–80 nm [12]	Unclear	Yes [13]	Yes [14]	Murine gall bladder epithelium [15]
CLIC/GEEC pathway endocytosis	Yes [16, 17]	Tubulovesicular, 40 nm width [18]	CTxB, CD44 [19]	Yes [16]	No [20]	COS, CHO cells [21]
Flotillin-dependent endocytosis	Unclear [22]	Unclear	Unclear	Yes [23]	Unclear	HeLa cells [24]
IL-2Rβ pathway endocytosis	Yes [25]	50–100 nm [25, 26]	IL-2Rβ [26]	Yes [27, 28]	Yes [29]	IARC 301.5, YT2C2, CIAC cells [30]
Arf6-dependent endocytosis	Yes [31]	60–200 nm	MHC-I, CD59 [32]	Yes [32]	Unclear	CHO cells [33]
Phagocytosis	Yes [34, 35]	0.5–3 μm [36–39]	Microbial pathogens	Yes [40, 41]	Yes [42]	Ranine phagocytes [43]
Fast endophilin-mediated endocytosis (FEME)	Yes [44]	Tubulo-vesicular, 100 nm–μm length	β1AR [44, 45]	Yes [44]	Yes [44]	BSC1, HEK293 cells [44]
Activity-dependent bulk endocytosis (ADBE)	Yes [46, 47]	150 nm	VAMP4 [48]	Yes [49]	Yes [50]	Murine cerebellar granule cells [51]
Ultrafast endocytosis (UFE)	Yes [52]	60–80 nm [52, 53]	Unclear	Yes [54]	Yes [52]	Nematode neurons [55]
Massive endocytosis (MEND)	No [56, 57]	< 100 nm [57]	Phospholemman, polypalmitoylated proteins [58]	Yes [59]	No [56, 57]	BHK, HEK293 cells [57]
Macropinocytosis	Yes [60, 61]	200 nm–20 μm	Non-selective	Yes [62, 63]	Unclear	Murine sarcoma cells [64]

Note that actin-dependency, dynamin-dependency, and canonical cargoes remain to be clarified for multiple pathways

Caveolae-dependent endocytosis is characterized by its requirement for the integral membrane protein caveolin-1 and a small number of adaptor proteins of the cavin family (four in mammals), as well as by its sensitivity to glycosphingolipid depletion [13, 71]. Caveolae, so named for their resemblance to caves, constitute small, flask-shaped membrane bulbs 50–100 nm in diameter and are enriched in vascular endothelial cells, epithelial cells, adipocytes, and fibroblasts [72, 73]. Trafficking of caveolar endosomes and delivery of their lumenal contents to organelles is poorly understood, in part because of overlap between cargoes sorted into the caveolae-dependent pathway and the CLIC-GEEC pathway [19].

Many proteins that are lipid-anchored to the outer leaflet of the plasma membrane, such as GPI-anchored aminopeptidases (GPI-APs), are endocytosed in uncoated, clathrin-independent carriers (CLICs) that are derived from the plasma membrane and enriched in large tubulovesicular structures called GPI-AP Enriched Early Endosomal Compartments (GEECs) [20, 21]. Endocytosis via the CLIC-GEEC pathway accounts for a significant proportion of internalized membrane and fluid-phase contents and in this respect resembles macropinocytosis, another form of CIE. Unlike macropinocytosis, however, CLIC-GEEC endocytosis is insensitive to amiloride inhibition [74].

The CLIC/GEEC pathway is initiated by membrane recruitment of GBF1, a guanine nucleotide exchange factor (GEF) for the GTP-binding protein ADP-ribosylation factor 1 (Arf1) [75]. Consequent to activation of Arf1 by GBF1, the Rho GTPase activating protein (GAP) ARHGAP10/21 is locally recruited and promotes the GTP cycling of Cdc42 [75]. Cdc42 dynamics at the membrane, in turn, regulate recruitment of downstream effectors that direct actin polymerization and promote the formation of CLICs. Enrichment of CLICs in GEECs is regulated by recruitment of GTPase regulator associated with focal adhesion kinase1 (GRAF1), a BAR-domain-containing protein that also negatively regulates Cdc42 via its Rho-GAP domain [18]. While the CLIC/GEEC pathway does not require Dynamin-1 or -2 for endocytosis of its cargoes, dynamin does associate with GEECs post-internalization [20].

Flotillin-dependent endocytosis is a form of CIE requiring flotillin (reggie) proteins, genes for which are highly conserved among metazoans [76]. Flotillins are characterized by N-terminal hydrophobic stomatin/prohibitin/flotillin/HflK/C (SPFH) domains shown to regulate membrane targeting in adipocytes and C-terminal flotillin domains necessary for oligomerization [77–79]. Flotillins associate with lipid rafts and generate membrane invaginations through mechansims that remain largely undefined [80]. The role of dynamin in flotillin-dependent endocytosis is also unclear, as is the mechanism governing cargo specificity. For these reasons, some have argued that flotillins may not characterize a distinct endocytic pathway at all but instead function as adaptors in other forms of CIE [81].

Many cytokine receptors are internalized via a cholesterol-sensitive pathway termed RhoA-dependent IL-2Rβ endocytosis for the receptor that historically first defined it. This form of CIE, which is initiated at the base of membrane protrusions, requires activation of the small GTPases RhoA and Rac1 as well as signaling through p21-activated kinases (Paks) [25, 26]. Two rounds of actin polymerization drive vesicular budding, maturation, and scission to form vesicles 50–100 nm in diameter [25, 26]. Dynamin has been shown to coordinate progressive recruitment of the actin effectors WAVE and N-WASP in IL-2Rβ endocytosis [25].

A variety of cell surface proteins, including those regulating nutrient and cholesterol homeostasis, are internalized in a manner requiring the small GTPase ADP-ribosylation factor 6 (Arf6) [82]. Arf6 GTP-loading in tubular endosomes promotes their recycling to the plasma membrane as well as the generation of actin-rich protrusions [31]. Mechanistically, Arf6-GTP activates phosphatidylinositol-4-phosphate 5-kinase, which in turn recruits additional signaling molecules to sites of active cytoskeletal arrangement to promote cargo internalization [83]. Intracellularly, Arf6-GDP associates with tubular early endosomes, then Rab5-positive sorting endosomes [84]. Subsequent trafficking events are regulated by the CME adaptor protein AP-2 [85]. Thus, Arf6 has been implicated in both CIE and dynamin-2-dependent CME [86].

Phagocytosis a form of CIE that involves the specific recognition and uptake of particles > 500 nm into membrane-derived vesicles known as phagosomes [87]. Phagocytosis is essential for development and tissue homeostastis, as well as a first line of defense against pathogens by innate immune cells [87]. Phagocytosis in these cells enables presentation of antigen to lymphocytes and activation of adaptive immune responses. Phagocytic target ligands are recognized by surface receptors that can be broadly classified as opsonic and non-opsonic. Opsonic receptors recognize foreign particles indirectly by binding host-derived opsonins [87]. Non-opsonic receptors include those that recognize pathogen-associated molecular patterns, as well as those that recognize apoptotic and necrotic cells [87]. Ligand binding initiates intracellular signaling cascades that activate the non-receptor protein tyrosine kinase Syk, generate phosphoinositide second messengers, and recruit activated Rho GTPases [87–89]. GTP-loaded Rho GTPases coordinate actin polymerization in phagocytic cups to engulf and internalize the particle [87].

Pinocytosis refers to non-specific endocytosis of contents dissolved in the fluid phase into vesicles of any size [90]. Micropinocytosis, the ingestion of fluid-phase contents into vesicles < 100 nm in diameter, is today and archaic term as it is now known to encompass a number of distinct endocytic pathways described elsewhere in this review. Macropinocytosis, however, refers to a distinct, evolutionarily-ancient, bulk form of endocytosis that is actin-mediated and leads to the generation of vesicles (macropinosomes) ranging in size from 200 nm to 5 µm in diameter [91]. All forms of fluid-phase endocytosis regulate cellular absorption of water, nutrients, and ions from the extracellular environment, though macropinocytosis regulates these processes at high throughput scale.

Macropinosomes are generated from large membrane ruffles and lamellapodial protrusions that either meet other protrusions at their distal margins or collapse back into the plasma membrane. In some respects macropinocytosis resembles phagocytosis, but unlike phagocytosis it is uniquely inhibited by amilorides, which block plasma membrane Na^+^/H^+^ exchangers [60, 92]. Macropinocytosis has been adapted for roles in diverse cellular processes including directed cell migration, feeding, and immune surveillance in antigen-presenting cells [91, 93, 94]. It is also exploited by some cancers to enable metabolic adaption and survival under nutrient-depleted conditions [95–97]. Growth factor-stimulated macropinocytosis has been shown to rely on sustained signaling through a Receptor Tyrosine Kinase (RTK)/PI3K signaling axis but RTK-independent, constitutive macropinocytosis has also been demonstrated [98].

Fast Endophilin Mediated Endocytosis (FEME) is a form of CIE regulated by the BAR-domain-containing protein endophilin, which has five paralogs in humans (A1, A2, A3, B1, and B2) [45]. FEME is a non-constitutive mode of endocytosis that occurs in response to activation of G-protein-coupled receptors (GPCRs) and cytokine receptors by their ligands. Activated receptors are sorted into pre-existing membrane clusters of endophilin that are rapidly (~ 5–10 s) internalized in tubulo-vesicular carriers 100 nm to microns in length that most closely resemble CLICs [44]. This form of CIE is dynamin-dependent, and, like many other forms of endocytosis, is regulated by phosphoinositide and kinase signaling [45]. In addition to its essential role in FEME, endophilin has been implicated in both IL-2Rβ endocytosis and CME; knock-down of endophilin has been shown to decrease the rate of IL-2Rβ internalization and to be required for the uncoating of CCVs in CME [44, 99].

Two high-capacity modes of CIE of special importance in neurons are Activity-Dependent Bulk Endocytosis (ADBE) and Ultrafast Endocytosis (UFE). Both are dynamin-dependent forms of CIE that, like FEME, are characterized by their rapidity. ADBE has been shown to internalize large patches of membrane and aid in the retrieval of synaptic vesicles (SVs) at central nerve terminals in response to high neuronal activity [46]. Mechanistically, ADBE requires interaction between dynamin and syndapin 1 to associate with N-WASP, an effector of actin nucleation and polymerization [100]. UFE occurs in response to more mild stimulation, 50–100 ms after propagation of an action potential, and enables the recycling of synaptic vesicle components, such as SNAREs and synucleins [52, 101]. Like FEME, endophilin has been implicated in regulation of UFE [102].

Lastly, Massive ENDocytosis (MEND) is a dynamin-independent form of CIE that does not require actin remodeling [57]. As the name suggests, MEND enables the internalization of very large membrane patches in response to metabolic stress, Ca^2+^ signaling, and other stimuli, in a manner driven by membrane phase separation [56, 103]. In this process, membranes of heterogenous lipid composition can partition into different nanodomains with intrinsic curvature, which facilitates endocytosis without actin remodeling.

## T cell endocytosis

CME and CIE facilitate a range of T cell specific functions, as summarized in Table 2. Chief among these are the regulation of plasma membrane immune receptors and signaling, including internalization and recycling of T cell antigen receptors (TCRs). Endocytic mechanisms are also critical for stable conjugate formation between T cells and APCs. They also enable trogocytic exchange of receptor complexes between individual T cells, as well as between T cells and APC. Lastly, TCR-stimulated uptake of key amino acids by macropinocytosis plays a critical role in promoting T cell anabolism and growth by sustaining activation of the mechanistic target of rapamycin complex 1 (mTORC1).

**Table 2:** Forms of endocytosis described in T lymphocytes

	Described in T cells	Function in T cells
Clathrin-dependent endocytosis	Yes	Plasma membrane receptor regulation [104, 105], TCR αβ endocytosis [106–108]
CLIC/GEEC pathway endocytosis	Yes	TCRζ endocytosis [109]
Flotillin-dependent endocytosis	Yes	TCR αβ recycling [110], conjugate formation with APCs [110]
IL-2Rβ pathway endocytosis	Yes	IL-2Rβ complex endocytosis [25, 111]
Arf6-dependent endocytosis	Yes	Conjugate formation with APCs [112]
Phagocytosis	Yes	Host defense/immune surveillance (γδ T cells) [113, 114], trogocytosis (TCR αβ T cells) [115]
Caveolae-dependent endocytosis	No	N/A
Macropinocytosis	Yes	mTORC1 activation and growth [98]
Fast endophilin-mediated endocytosis (FEME)	Yes	IL-2Rβ complex endocytosis [44]
Activity-dependent bulk endocytosis (ADBE)	No	N/A
Ultrafast endocytosis (UFE)	No	N/A
Massive endocytosis (MEND)	No	N/A

### Plasma membrane immune receptor and ligand regulation

Endocytosis of plasma membrane receptors and the trafficking, recycling, and targeted degradation of receptor components are integral to many cellular responses, including those of T cells. Both CME and CIE pathways have been shown to regulate plasma membrane immune receptors in both TCR αβ and γδ T cells.

The immune checkpoint protein CTLA-4, which negatively regulates TCR αβ T cell activation by out-competing CD28 and trans-endocytosing its ligands CD80 and CD86, is constitutively internalized by CME [104, 105]. This occurs in a ligand- and dynamin-independent manner and results in both recycling to the cell surface and trafficking to lysosomes for degradation. Constitutive, ligand-independent internalization continues even as CTLA-4 surface expression is upregulated during T cell activation.

In thymus-dependent humoral immune responses, transient expression of the transmembrane glycoprotein CD40-L on CD4^+^ TCR αβ T cells provides an essential, contact-dependent, co-stimulatory signal to cognate B cells. CD4^+^ T cell CD40-L binding to CD40 on B cells initiates an intracellular signaling cascade that promotes the generation of class-switched, high-affinity antibodies, as well as the establishment of B cell memory and differentiation into long-lived plasma cells. In addition to the well-established transfer of CD40-L from Tfh cells to cognate B cells via an unknown exocytic mechanism, down-modulation and lysosomal degradation of plasma membrane CD40-L has also been shown to occur in T cell tumor lines [116]. Endocytosis of CD40-L in these cells requires actin polymerization, though its dependence on clathrin and dynamin have not been established.

By contrast, the rapid internalization of IL-2R complexes from the surface of activated TCR in αβ T cells has been shown to occur by CIE [111]. IL-2Rβ endocytosis was first demonstrated to be clathrin-independent in studies employing dominant-negative mutants of the essential clathrin coated pit and vesicle component Eps15 [111]. Endocytosis of IL-2Rβ complexes in these experiments occurred normally in the absence of CME as measured by transferrin uptake. In addition to dynamin, IL-2Rβ internalization requires the cytoplasmic tail of the component γ_c_ chain, as well as both the catalytic activity and p85 regulatory subunit of PI3K [117, 118]. The constituent subunits of the receptor partition into different compartments soon after internalization, with the comparatively stable α chain confined to transferrin-positive recycling endosomes (suggesting partial utilization of the CME pathway) whereas the β and γ_c_ chains are sorted into late endosomes and thereafter targeted to lysosomes for degradation [119]. The proteasome has also been shown to be important, not for the initial phase of IL-2Rβ endocytosis but for its continuance and lysosomal targeting of the β and γ_c_ subunits [120]. The co-localization of endophilin with IL-2Rβ vesicular cargoes in the human T cell line Kit255, as well as the specific diminution of IL-2Rβ internalization in cells depleted of endophilin, implicate FEME as a mechanism of IL-2Rβ endocytosis [44]. Whether these represent two distinct endocytic pathways or simply utilize shared machinery remains to be clarified.

WC1 proteins, transmembrane glycoproteins of the scavenger receptor cysteine-rich family, are co-receptors of the TCR in γδ T cells. They are thought to function as bacterial pattern recognition receptors that regulate cell activation by co-ligation with the γδ TCR [121]. WC1 is endocytically down-regulated in response to non-specific stimulation by phorbol 12-myristate 13-acetate (PMA) [122]. It has been shown in Jurkat T cells that a dileucine motif in the cytoplasmic domain of WC1 regulates co-receptor endocytosis induced by PMA [121]. In this system, sustained co-ligation of the TCR and a transmembrane fusion protein consisting of the CD4 extracellular domain joined to the WC1 transmembrane and cytoplasmic domains enhanced T cell activation, as measured by elevated IL-2 production [121]. Like the CD3γ, CD3δ, and CD4 intracellular domains, the proximal cytoplasmic tail of WC1 contains a [DE]XXXL[LIM] dileucine motif known to bind to the adaptor protein (AP-2) components of CCPs and CCVs [121]. The presence of this motif on WC1 family proteins suggests that endocytosis of the WC1 co-receptor in γδ T cells is regulated by CME.

### Endocytosis of the TCR in αβ T cells

In the absence of stimulation, non-engaged TCRs are constitutively internalized by dynamin-dependent CME and recycled back to the cell surface [106]. Endocytosis of engaged TCRs, on the other hand, occurs by both CME and CIE [110]. Mechanosensory cues appear to play a role in dictating which mode predominates: TCR triggering with soluble anti-CD3 antibodies promotes internalization by CME, whereas triggering by anti-CD3 immobilized on plastic promotes CIE of engaged TCRs [106].

The clathrin-dependent pathway requires dynamin and is similarly regulated by a CD3γ dileucine endocytosis motif. Endocytosis and signaling from engaged TCRs is tightly coupled, as it is for other signaling components of TCR microclusters, such as LAT, ZAP-70, and SLP-76. It has been shown in CD4^+^ and CD8^+^ human T cell lines that the Src family kinase Lck, a key component of the T cell signalosome, promotes CME of the TCR upon receptor engagement and lysosomal degradation [107, 123]. It does so by inducible phosphorylation of tyrosine residues on the clathrin heavy chain (CHC) which interact with the clathrin light chain to regulate cage assembly [107]. Basal Lck phosphorylation of the CHC also plays a role in constitutive endocytosis of the TCR, as unstimulated cells deficient in Lck exhibit no TCR internalization [107]. Another Src family kinase that regulates proximal TCR signaling, Fyn, also promotes CME of the TCR, since human T cell lines deficient in CD45, and therefore unable to activate Lck or Fyn, exhibit less internalization than those deficient in Lck alone [123, 124].

An adaptor protein critical for the early-stage assembly of CCPs, the FCH domain only 1 (FCHO1) protein, also plays a critical role in CME of engaged TCRs. First identified by whole exome sequencing in human patients with combined immunodeficiency, loss-of-function mutations in FCHO1 profoundly impair ligand-induced TCR clustering and endocytosis [108, 125]. FCHO1 deletion in Jurkat T cells recapitulates this phenotype and can be rescued by expression of wild-type FCHO1 [108].

Another adaptor critical for CME of engaged TCRs is the cytoplasmic protein intersectin 2, which has been shown to promote the translocation of Cdc42 and its effector Wiskott-Aldrich Syndrome protein (WASP) to CCVs in Jurkat cells [126]. Intersectin 2 also activates Cdc42 by its Dbl homology (DH)/RhoGEF domain. Overexpression of intersectin 2 in Jurkats substantially increases TCR internalization whereas expression of an intersectin 2ΔDH construct markedly reduced it [126]. In this way, intersectin 2 may link the machinery of actin polymerization with that of CME in T cells.

Sustained TCR signaling is required for full cell activation and this depends critically on the delivery of signaling-competent, TCR-laden recycling endosomes to the immunological synapse (IS) [127]. This mechanism compensates for the activation-induced down-modulation of engaged receptors, which is required for serial triggering of receptors and desensitization of stimulated cells. It has also recently been shown that activated TCR-CD3ζ complexes internalized by CME continue to signal from endosomes positive for insulin responsive aminopeptidase (IRAP) and Syntaxin 6, and that this activity is required for efficient anti-tumor T cell responses [128].

Interestingly, selective triggering of the TCR complex has been shown to also cause the concomitant downregulation of non-engaged TCRs in a manner regulated by protein kinase C θ (PKCθ) and the CME adaptor AP-2 [129]. Bystander TCR downmodulation that occurs concomitantly with TCR ligation, however, uniquely requires protein tyrosine kinase (PTK) activity [106].

The clathrin-independent pathway of TCR endocytosis uniquely utilizes the Rras subfamily GTPase TC21. TC21 promotes internalization by a mechanism reliant on the small GTPase RhoG, previously implicated in both phagocytosis and caveolar endocytosis [115, 130, 131].

The CLIC-GEEC pathway of CIE has also been implicated in TCR endocytosis in activated Jurkat T cells. In this system, CD3 triggering resulted in TCRζ accumulation in tubular invaginations of the plasma membrane that are shaped by actin polymerization downstream of the Rho GTPase Cdc42 [109]. The BAR domain-containing protein GRAF1 is recruited to these structures, where it promotes Cdc42 GTP hydrolysis via its GAP domain. These tubular invaginations mature into endocytic vesicles that show co-localization of the internalized TCR with cholera toxin B and CD44, established cargoes of the CLIC-GEEC pathway [109].

A number of proteins associated with CIE pathways appear to regulate TCR endocytosis through their effects on endocytic trafficking. The actin-binding protein HIP-55 is recruited to the IS in activated Jurkat cells and associates with early endosomes and dynamin [132]. In these cells, HIP-55 expression promotes basal and ligand-dependent TCR down-modulation, most likely by interfering with receptor recycling [132].

Members of the EPS15 Homology Domain-containing (EHD) family of endocytic traffic regulators are expressed in murine CD4^+^ T cells and have also been implicated in the regulation of cell surface receptors. CD4^+^ T cells from conditional knockout EHD1/3/4 mice exhibit reduced proliferation and IL-2 secretion in response to antigen stimulation in vitro, as well as impaired TCR recycling, and enhanced lysosomal degradation of TCR components [133]. Support for a role in these processes comes from the association of EHD proteins with Rab effector proteins, which regulate endocytic trafficking [133].

Membrane-organizing flotillin proteins incorporate into pre-assembled signaling platforms that asymmetrically localize to one pole in hematopoietic cells, including T cells [134]. Immediately upon internalization, engaged TCRs are incorporated into a stable, mobile endocytic network defined by flotillins [110]. Consistent with the idea that flotillins may function as adaptors for other endocytic pathways, as opposed to demarcating a distinct, bona fide form of endocytosis, they are not required for internalization of engaged TCRs. Like EHD proteins, flotillins may regulate TCR surface expression by promoting endocytic recycling. Flotillins are required for the trafficking of downmodulated TCRs to Rab5-positive sorting endosomes, from Rab5- to Rab11a-positive recycling endosomes, and their recycling to the IS [110, 135].

### Arf6-mediated endocytosis and flotillins in APC conjugate formation

The formation of stable conjugates between T cells and APCs requires Arf6, Rab22, and flotillins [112]. Expression of a constitutively-active form of Arf6 in Jurkat T cells inhibits endocytosis of MHC class I, and causes other cargoes important for IS formation, such as CD4 and LFA-1, to accumulate in enlarged, Arf6-positive vacuoles [112]. Consequently, conjugate formation with APCs is impaired. In addition to Arf6, the GTPase Rab22 (a.k.a. Rab22a) is also required to form stable Jurkat–Raji (B) cell conjugates as expression of a dominant-negative form of it (Rab22S19N) is sufficient to impair their formation [112]. Additionally, Jurkat T cells deficient in flotillin1/2 show are unable to form stable conjugates with Raji cells, demonstrating a requirement for flotillin proteins in this process as well [110].

### Phagocytosis in TCR γδ T cells

Previously thought to be limited to cells of the myeloid lineage, it is now known that human peripheral γδ T cells not only have phagocytic capabilities but can act as “professional” phagocytes in that they are capable of presenting processed antigen on MHC class II to TCR αβ T cells [113, 114]. Indeed, TCR γδ T cells can ingest entire bacteria, such as *L. monocytogenes* and *E. coli* [113, 114]. Presumably the maturation of phagosomes in these cells resembles and depends on the same machinery as other professional phagocytes (e.g., Rab5/7, RILP, etc.) though very little is currently known about this.

### Trogocytosis

Trogocytosis refers to the exchange of intact membrane patches between cells. While not, strictly-speaking, a form of endocytosis, in vitro studies have suggested a mechanism with qualitative similarity to that of phagocytosis. An increasing body of evidence suggests not only that T cell trogocytosis is a ubiquitous phenomenon in vivo, but that it constitutes an important mechanism of intercellular communication and immune modulation [136–139]. Trogocytosis has even been shown to convey novel functional capabilities from one cell type to another through the acquisition of membrane-associated molecules [136, 140].

In Jurkat T cells, TCR-mediated trogocytic uptake of peptide:MHC complexes from antigen-presenting cells requires TC21 (Rras2) and the phagocytosis-associated GTPase RhoG [115, 141]. In CD4^+^ TCR αβ T cells, trogocytic exchange of peptide:MHC complexes has been shown to influence T effector cell polarization [138]. When stimulated by murine fibroblasts and peptide-pulsed bone marrow-derived dendritic cells expressing peptide:MHC complexes, trogocytosis-positive CD4^+^ T cells activated the transcription factor GATA-3 and produced IL-4 both in vitro and in vivo, consistent with Th2 polarization [138]. The mechanism responsible for this polarization remains to be elucidated, though it may relate to the strength and duration of TCR stimulation.

Even more remarkably, virus-specific CD8^+^ cytotoxic T lymphocytes (CTLs) are capable of transferring their TCRs via trogocytosis to recipient CTLs of different clonotypic specificity [142]. Acquisition of donor TCRs confers the ability to recognize additional antigen and enables expansion of virus-specific clones independent of proliferation [142]. On the other hand, the detrimental potential of trogocytosis-mediated T cell plasticity is demonstrated by a recent study by Haimeh et al. examining chimeric antigen receptor (CAR) T cell responses in a mouse leukemia model. In this work, trogocytic acquisition of target antigen by CAR T cells not only reduced target density on tumor cells but promoted “fratricidal” (mutual) CAR T cell killing and exhaustion [143].

### Macropinocytosis

**Fig. 1:**
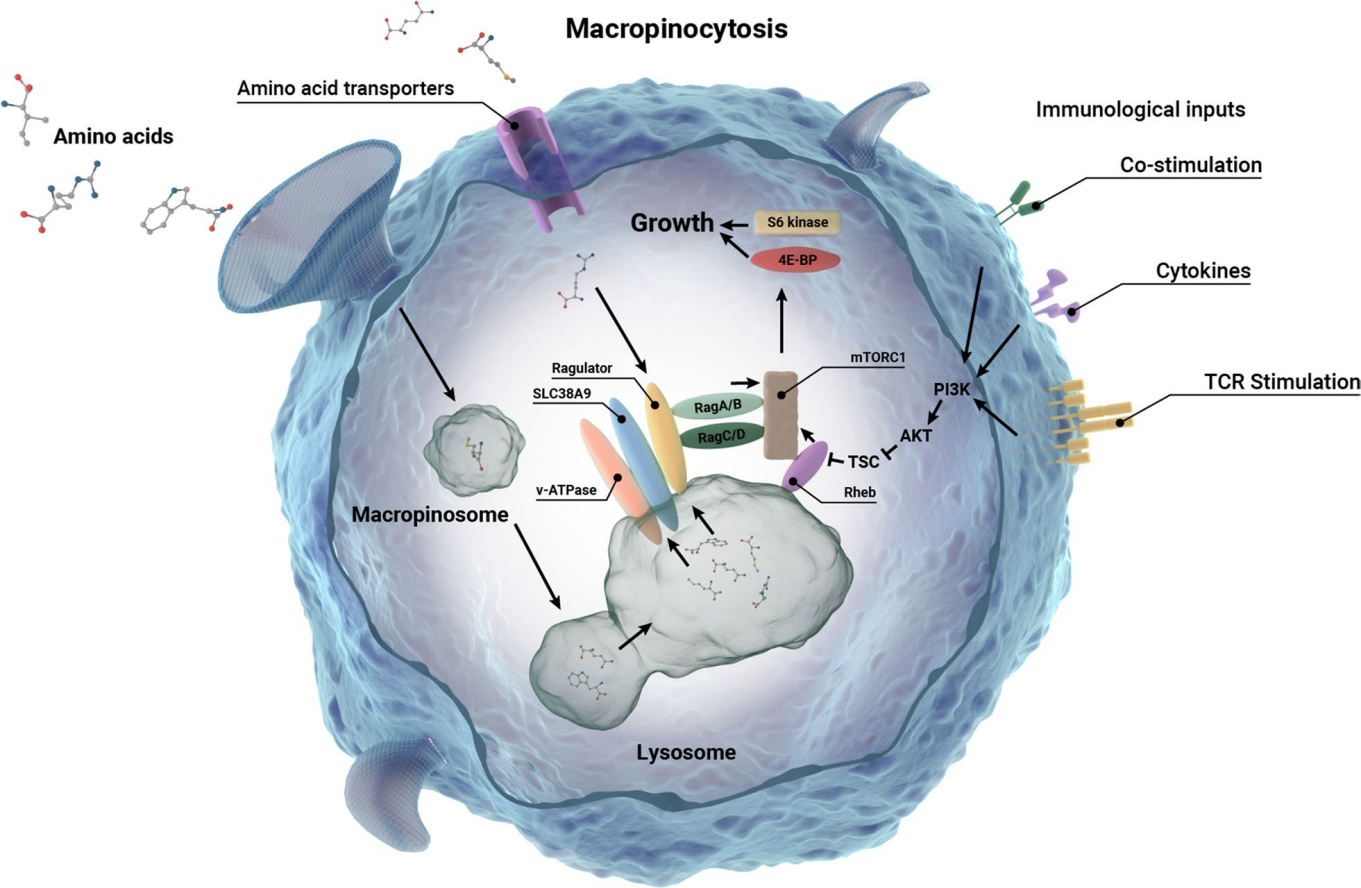
Macropinocytosis in T cells. T cells use macropinocytosis to deliver extracellular amino acids (LQRS) to lysosomes necessary for the activation of mTORC1 that drives T cell growth (see text for details)

Macropinocytosis has been described in both murine and human primary TCR αβ T cells. It is a constitutive activity in naïve T cells that is upregulated several-fold in response to stimulation and activation [98]. One established function of macropinocytosis in T cells is to internalize and deliver free amino acids (as opposed to protein) obtained from the extracellular space to the lysosome [98]. Intralumenal amino acids signal, most likely through a membrane transceptor, to promote the activation of the mechanistic target of rapamycin complex 1 (mTORC1), a central regulator of anabolism and cellular growth [98]. In this way, macropinocytosis promotes nutrient acquisition and growth signaling in T cells preparing to undergo clonal expansion (Fig. 1).

It is interesting to speculate on the reasons why TCR-stimulated macropinocytosis is required for optimal activation of naïve CD4^+^ and CD8^+^ T cells. After all, most if not all of the forms of endocytosis previously described are sufficient to transport amino acids and a wide variety of amino acid transporters are expressed in these cells. One possibility is that transport through other endocytic pathways does not license lysosomal delivery of internalized cargoes. Some pathways clearly are sufficient for this purpose: the IL-2-IL2R complex has been shown to deliver IL-2 to primary T cell lysosomes, albeit with a required stopover at the proteasome beforehand [120].

A more likely explanation is that while naïve T cells express abundant plasma membrane amino acid transporters, their flux capacity is insufficient to meet the demand of T cell activation. Activation requires dramatic increases in amino acid and glucose uptake to enable previously metabolically quiescent cells to upregulate aerobic glycolysis and glutaminolysis. Consequently, TCR signaling and co-stimulation substantially increases expression and plasma membrane localization of GLUT1 and amino acid transporters such as LAT1, SNAT-1, and SNAT-2 [144]. It’s possible that lysosomal import of cytoplasmic amino acids requires an adaptor that is not abundantly expressed in naïve or nascently-activated T cells. Such is the case for the adaptor LAPTM4b, which recruits the LAT1 transporter to the lysosome and is required for mTORC1 activation in HeLa cells [145].

Another possibility is that, bulk acquisition of amino acids by macropinocytosis may more rapidly or efficiently activate lysosomal mTORC1 complexes than the transcription, translation, and membrane-targetting of activation-induced transporters will permit. The nearly indetectable levels of LAT1 protein in naïve human T cells support this hypothesis [146].

A last (and not mutually exclusive) possibility is that maximal mTORC1 activation in these cells requires not *only* an intralysosomal amino acid sufficiency signal but also a second signal conveyed by cytoplasmic amino acid sensors like Sestrin2 and CASTOR1. In this way, mTORC1 may function like an AND gate, sensitive to both intracellular and extracellular amino acid signal inputs, where a concentration gradient exists between lysosomal and cytoplasmic amino acid pools.

## Conclusions

The term endocytosis encompasses a range of diverse cellular mechanisms for regulating membrane composition and internalizing contents from the extracellular space. While CME has been shown to be the principal housekeeping mode of endocytosis in resting cells, multiple, overlapping modes of CIE have more recently been described in most eurkaryotic cell types. In T cells both CME and CIE are employed to facilitate cell-specific functions, including regulation of T cell receptor internalization and signaling, interaction with APCs, effector cell polarization, and nutrient acquisition. With the exception of caveolae-dependent endocytosis and several high capacity modes of endocytosis that appear to be specific to neurons, flux through CIE pathways regulates and enables a range of critical T cell functions.

## Data Availability

Not applicable.

## Abbreviations

ADBE: Activity-dependent bulk endocytosis
APC: Antigen-presenting cell
Arf1/6: ADP-ribosylation factor 1/6
CASTOR1: Cytosolic arginine sensor for mTORC1 subunit 1
CCP: Clathrin coated pit
CCV: Clathrin coated vesicle
CIE: Clathrin-independent endocytosis
CLIC/GEEC: Clathrin-independent carrier/GPI-AP-enriched early endosomal compartment
CME: Clathrin-mediated endocytosis
CTL: Cytotoxic T lymphocyte
EHD: EPS15 Homology Domain-containing
FCHO1: FCH domain only 1
FEME: Fast endophilin-mediated endocytosis
GRAF1: GTPase regulator associated with focal adhesion kinase1
LAT1: Large amino acid transporter 1
LFA-1: Lymphocyte function-associated antigen-1
MEND: Massive enocytosis
MHC: Major histocompatibility complex
mTORC1: Mechanistic target of rapamcyin
Paks: P21-activated kinases
PKCθ: Protein kinase C θ
PMA: Phorbol 12-myristate 13-acetate
RILP: Rab-interacting lysosomal protein
RTK: Receptor tyrosine kinase
SNAT-1/2: Sodium-coupled neutral amino acid transporter-1/2
SVs: Synaptic vesicles
TCR: T cell receptor
Tfh: T follicular helper
UFE: Ultrafast endocytosis
WASP: Wiskott–Aldrich syndrome protein
WAVE: WASP-family verprolin homologous protein
